# RNA Sequencing of Collecting Duct Renal Cell Carcinoma Suggests an Interaction between miRNA and Target Genes and a Predominance of Deregulated Solute Carrier Genes

**DOI:** 10.3390/cancers12010064

**Published:** 2019-12-24

**Authors:** Sven Wach, Helge Taubert, Katrin Weigelt, Nora Hase, Marcel Köhn, Danny Misiak, Stefan Hüttelmaier, Christine G. Stöhr, Andreas Kahlmeyer, Florian Haller, Julio Vera, Arndt Hartmann, Bernd Wullich, Xin Lai

**Affiliations:** 1 Department of Urology and Pediatric Urology, University Hospital Erlangen, Friedrich-Alexander University Erlangen-Nürnberg, 91054 Erlangen, Germany; sven.wach@uk-erlangen.de (S.W.); Katrin.Weigelt@uk-erlangen.de (K.W.); Andreas.Kahlmeyer@uk-erlangen.de (A.K.); Bernd.Wullich@uk-erlangen.de (B.W.); 2 Institute of Molecular Medicine, Section for Molecular Cell Biology, Faculty of Medicine, Martin Luther University Halle-Wittenberg, 06120 Halle, Germany; nora.hase@medizin.uni-halle.de (N.H.); marcel.koehn@medizin.uni-halle.de (M.K.); danny.misiak@medizin.uni-halle.de (D.M.); stefan.huettelmaier@medizin.uni-halle.de (S.H.); 3 Department of Pathology, University Hospital Erlangen, Friedrich-Alexander University Erlangen-Nürnberg, 91054 Erlangen, Germany; Christine.Stoehr@uk-erlangen.de (C.G.S.); Florian.Haller@uk-erlangen.de (F.H.); arndt.hartmann@uk-erlangen.de (A.H.); 4 Laboratory of Systems Tumor Immunology, Department of Dermatology, University Hospital Erlangen, FAU Erlangen-Nürnberg, 91054 Erlangen, Germany; julio.Vera-Gonzalez@uk-erlangen.de

**Keywords:** collecting duct carcinoma, RNA sequencing, solute carrier proteins

## Abstract

Collecting duct carcinoma (CDC) is a rare renal cell carcinoma subtype with a very poor prognosis. There have been only a few studies on gene expression analysis in CDCs. We compared the gene expression profiles of two CDC cases with those of eight normal tissues of renal cell carcinoma patients. At a threshold of |log2fold-change| ≥1, 3349 genes were upregulated and 1947 genes were downregulated in CDCs compared to the normal samples. Pathway analysis of the deregulated genes revealed that cancer pathways and cell cycle pathways were most prominent in CDCs. The most upregulated gene was *keratin 17*, and the most downregulated gene was *cubilin*. Among the most downregulated genes were four solute carrier genes (*SLC3A1*, *SLC9A3*, *SLC26A7*, and *SLC47A1*). The strongest negative correlations between miRNAs and mRNAs were found between the downregulated miR-374b-5p and its upregulated target genes *HIST1H3B*, *HK2*, and *SLC7A11* and between upregulated miR-26b-5p and its downregulated target genes *PPARGC1A*, *ALDH6A1*, and *MARC2*. An upregulation of HK2 and a downregulation of PPARGC1A, ALDH6A1, and MARC2 were observed at the protein level. Survival analysis of the cancer genome atlas (TCGA) dataset showed for the first time that low gene expression of *MARC2*, *cubilin*, and *SLC47A1* and high gene expression of *KRT17* are associated with poor overall survival in clear cell renal cell carcinoma patients. Altogether, we identified dysregulated protein-coding genes, potential miRNA-target interactions, and prognostic markers that could be associated with CDC.

## 1. Introduction

Collecting duct renal cell carcinoma (CDC; also known as Bellini duct carcinoma, collecting duct carcinoma of the kidney) is a very rare (approximately 1–2%) but also very aggressive renal cell carcinoma with a median survival time of 11 months [1,2,3]. Tumors concerning the collecting duct were first described independently by Mancilla-Jimenez et al. and by Cromie et al. [4,5]. The putative cell of origin is in the distal convoluted tubules, a segment between the proximal tubules and the distal part of the nephron [6]. There are several cytogenetic abnormalities known, i.e., mostly loss of 11, 6p, 8p, 9p, and 21q and the Y chromosome as reviewed in [3]). However, there have been only a few reports about chromosomal aberrations, mutations in CDCs, and RNA expression changes [6,7,8]. Pal et al. identified clinically relevant genomic alterations mostly in genes *NF2*, *SETD2*, *SMARCH1*, and *CDKN2A* (29% to 12%) but also in 6% of genes *PIK3CA*, *PIK3R2*, *FBXW7*, *BAP1*, *DNMT3A*, *VHL*, and *HRAS* [7]. Furthermore, amplifications of *ERBB2* and genomic alterations of *SMARCB1* have been described [7]. Malouf et al. performed the first transcriptomic analysis of CDC and compared it with upper tract urothelial carcinomas (UTUCs) [6]. In addition to the finding that the CDC transcriptome is unique and clustered with that of clear cell renal cell carcinoma (ccRCC) patients rather than UTUC patients, the authors compared CDCs with UTUCs and identified *CDH6* and *POU3F3* as the top upregulated genes and *GATA3*, *TP63*, *KRT17*, *KRT7*, *KRT20*, *UPK2*, *UPK1A*, and *UPK3A* as the top downregulated genes in CDCs [6]. Based on the transcriptomic signature, they concluded that CDC is a disease characterized by metabolic and immunogenic aberrations. Wang et al. reported in a combined whole-exome sequencing and transcriptome sequencing study of CDC that many single nucleotide variations in cancer census genes, but also deletions of *CDKN2A*. In addition, RNA expression changes in members of the solute carrier (SLC) family, such as overexpression of *SLC7A11* (*cystine transporter*, *xCT*), have been reported [8].

Promising treatment schemes for metastasized renal cell carcinoma have been reported [9,10], but they mostly concern ccRCC, and there is still no specific therapy for CDC. However, there are treatment suggestions for metastatic CDC, i.e., first-line therapy with a combination of chemotherapy (gemcitabine) plus cisplatin/carboplatin, and second-line therapy as a targeted therapy [2,3]. Suggestions to treat CDC patients with drugs that target solute carriers, such as SLC7A11 or SLC6A7, have been made previously [11]. However, further molecular characterization of CDC is needed to better understand its tumor biology and to identify potential therapeutic targets.

In our study, we performed RNA transcriptome sequencing of two CDC cases and eight normal tissues in an effort to better characterize this rare tumor entity. We investigated differences in gene expression and sought to describe single nucleotide variation patterns and utilized pre-miRNA expression data in an effort to identify potentially regulated target proteins. Based on the finding of a predominance of gene expression changes in solute carriers in CDC, and our previous results concerning miRNAs and their target gene expression as biomarkers in urologic cancers, we focused our analysis on these two research fields. We found that several solute carrier genes are significantly dysregulated in CDC. In addition, we showed that the low expression of *SLC47A1* leads to poor survival of clear cell renal cell carcinoma patients, suggesting it as a prognostic marker for CDC.

## 2. Results

### 2.1. RNA Sequencing Revealed Up- and Downregulated Genes

**Figure 1:**
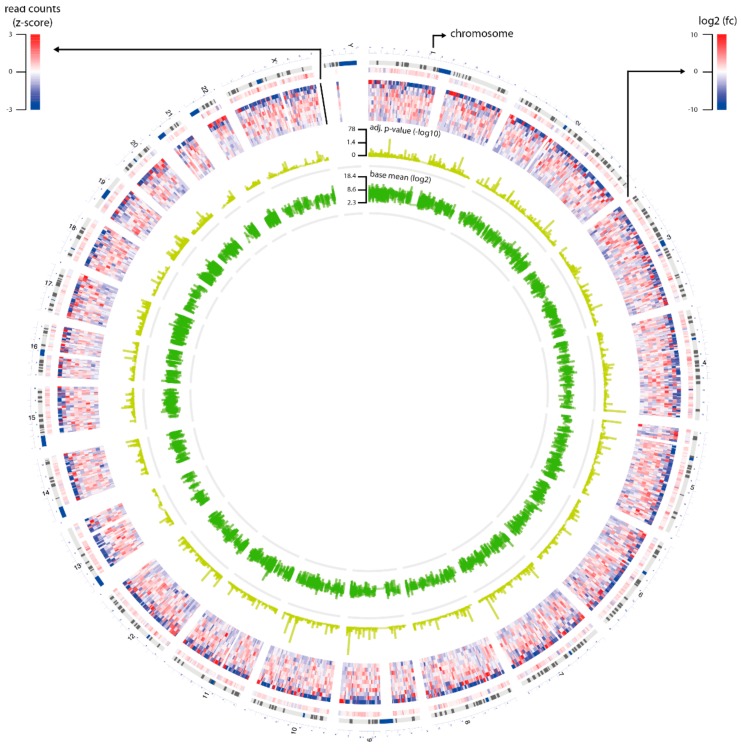
Overview of RNA sequencing data. The circus plot shows statistics of the genes (*n* = 16,672) that were selected for differential gene expression analysis. The plot contains four circles: Layer 1 is log2fold-change of the genes; Layer 2 is read counts of the genes that were transformed to z-score; Layer 3 is adjusted *p*-value of the genes that were transformed by–log10; and Layer 4 is the base mean of the genes that were transformed by log2.

**Figure 2:**
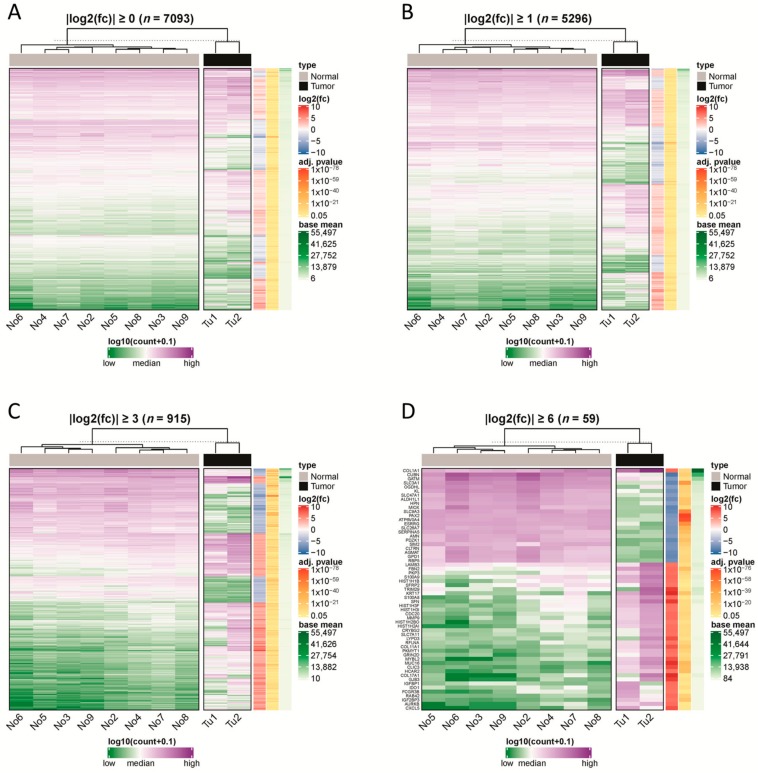
Hierarchical clustering of samples using differentially expressed protein-coding genes. We used different-fold changes as a threshold to filter significantly differentially expressed protein-coding genes (adjusted *p*-value ≤ 0.05). Read counts of the genes were used to cluster normal and tumor samples based on their Euclidian distance. In addition, we annotated the selected genes with their log2fold-change, adjusted *p*-value and base mean of their read counts. The thresholds used were (**A**) |log2fold-change| ≥0, (**B**) |log2fold-change| ≥1, (**C**) |log2fold-change| ≥3, and (**D**) |log2fold change| ≥6.

RNA transcriptome sequencing was performed for two CDC cases and eight histologically normal tissue samples (Figure 1). Upon analyzing the read counts, a total of 7093 coding genes were detected as being significantly deregulated between the CDC and normal tissue samples (*p* < 0.05). After hierarchical clustering, it became evident that the two CDC samples formed a cluster that was very distinct from the normal tissue samples (Figure 2). Interestingly, the normal tissue samples, which were also derived from tumor-bearing kidneys of different entities, did not show any tendency to cluster according to their corresponding tumor entity, which strongly suggests the absence of any field effect. For filtering purposes, the differential expression measure was log2 transformed. Application of a |log2fold change| ≥1 cutoff revealed that 1,947 genes were downregulated and 3,349 genes were upregulated in CDCs vs. normal samples (Table S1). The clustering results were comparable to the result without filtering.

After applying a |log2fold change|≥3 cutoff, only 316 genes were detected as downregulated and 599 genes as upregulated in the CDC compared to the normal tissue samples (Table S1). The clustering analysis still demonstrated a clear distinction between the two CDC samples on one side and the normal samples on the other side. Among the 915 significantly deregulated genes with a |log2fold change| ≥3 were 15 downregulated and 11 upregulated SLC genes, which comprised 2.8% of all genes.

With even more strict filtering criteria for differential expression, |log2fold change| ≥6 (which corresponds to a 64-fold difference), we could still identify 57 deregulated genes, of which 22 genes were downregulated and 37 genes were upregulated in CDC compared to normal samples (Table S1). Again, the CDC samples formed a cluster distinct from the normal tissue samples, but subtle differences in their gene expression patterns were more obvious. Again, the normal tissue clustered closer together. Of note, among the upregulated genes were five histone 1 genes (*HIST1H2BO*, *HIST1H3I*, *HIST1H3F*, *HIST1H1B*, and *HIST1H2AI*) and three collagen genes (*COL1A1*, *COL11A1*, and *COL17A1*). Remarkably, among the |log2fold change| ≥6 deregulated genes, four solute carrier genes were found to be downregulated (*SLC3A1*, *SLC9A3*, *SLC26A7*, and *SLC47A1*), and one solute carrier gene was found to be upregulated (*SLC7A11*). The fact that a total of 8.8% of the genes with a |log2fold change| ≥6 belong either to histone 1 genes or to SLC genes is very remarkable. We will return to *SLC7A11* and *SLC47A1* later in our study.

On a global scale, the top downregulated gene in the CDC samples compared to the normal tissue samples was *cubilin* (*CUBN*) (Table S1), which is highly expressed in normal renal proximal tubules [12]. The top upregulated gene in the CDC samples was *keratin 17* (*KRT17*; Table S1). KRT17 is an intermediate filament protein rapidly induced in wounded stratified epithelia. It regulates cell growth and stimulates the Akt/mTOR pathway and glucose uptake [13,14,15].

### 2.2. Pathway Analyses

To gain a more comprehensive insight into the signaling pathways that are potentially affected in CDC, we first applied a |log2fold change| ≥1 cutoff and then performed a gene set enrichment analysis with pathways derived from three independent databases, KEGG, WikiPathway, and Reactome (Table S2). After mapping against the KEGG database, the terms “pathways in cancer”, “cell cycle”, and “small cell lung cancer” were found among the top 10 affected pathways. When mapping against the WikiPathway database, the terms “retinoblastoma in cancer”, “integrated pancreatic cancer pathway”, and “cell cycle” were found among the top 20 enriched pathways. Finally, the Reactome database revealed that “collagen” and “mitotic cell cycle” were among the top 10 enriched pathways. In summary, using different pathway databases, we were able to demonstrate that the genes deregulated in CDC are enriched in distinct cancer-related signaling pathways and pathways affecting cell cycle regulation.

### 2.3. Investigation of SNPs and Mutations

In an effort to identify a possible association between single nucleotide variants and the occurrence of CDC, we screened the RNA transcriptome sequencing data for single nucleotide variations between the CDC and the normal samples (Table S3). The identified variations were first mapped against the NCBI SNP database to identify known variants with accession numbers. To define the potential clinical relevance, every identified variant was queried against the NCBI ClinVar database to check whether the variations were pathogenic, likely pathogenic, or confer sensitivity or drug response [16]. However, none of the identified variations were indicated to have these features.

### 2.4. Correlations of miRNAs and Target mRNA Expression

**Figure 3:**
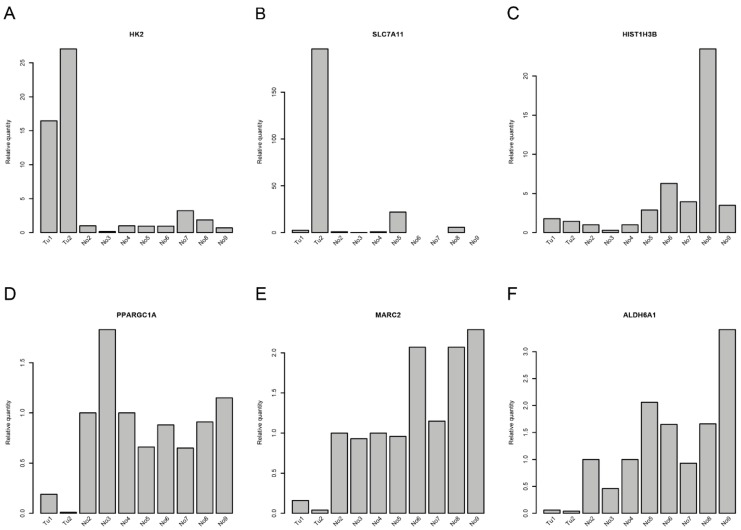
Quantitative RT-PCR for deregulated genes in collecting duct renal cell carcinoma (CDC). Gene expression of (**A**) *HK2*, (**B**) *SLC7A11*, (**C**) *HIST1H3B*, (**D**) *PPARGC1A*, (**E**) *MARC2*, and (**F**) *ALDH6A1* in the samples that were used for RNA sequencing.

Correlations between miRNAs and their corresponding target genes can reveal regulatory mechanisms in tumor biology. From the RNA transcriptome sequencing data, we were able to extract information about the expression of pre-miRNAs. Correlations between the mature miRNAs that could be processed from the assessed pre-miRNAs and target mRNAs are shown in Table 1 and Table S4. The strongest correlations between miRNAs and mRNA expression levels were found for miR-374b-5p and miR-26b-5p and their respective target genes (Table 1 and Table S4). Whereas miR-374b-5p was downregulated (1.65-fold; adjusted *p*-value = 0.155) in CDC samples, miR-26b-5p (1.55-fold; adjusted *p*-value = 0.021) was upregulated in CDC samples compared to normal samples. Accordingly, the target genes of miR-374b-5p were upregulated, and those of miR-26b-5p were downregulated in CDC samples. The associations derived from transcriptome sequencing data were further validated in the original RNA preparations by qRT-PCR. We could verify the upregulation of the target genes of miR-374b-5p, i.e., *HK2* and, in one CDC sample, *SLC7A11*, but not *HIST1H3B* (Figure 3A–C). Furthermore, the downregulation of the target genes of miR-26b-5p, i.e., *PPARGC1A*, *ALDH6A1*, and *MARC2*, could be validated (Figure 3D–F). Interestingly, *SLC7A11* is a predicted target of both miRNAs, raising the possibility of competitive binding of both miRNAs to their respective binding sites in the 3’UTR of the *SLC7A11* gene. There are four potential binding sites for miR-26b-5p and four potential binding sites for miR-374b-5p in the 3’UTR of the *SLC7A11* gene. However, the closest distance between any binding sites of these two miRNAs is 55 nt (Table S5), which argues against a competition between these two miRNAs for binding sites in the 3’UTR of the *SLC7A11* gene.

### 2.5. Protein Expression of miRNA Target Genes

**Figure 4:**
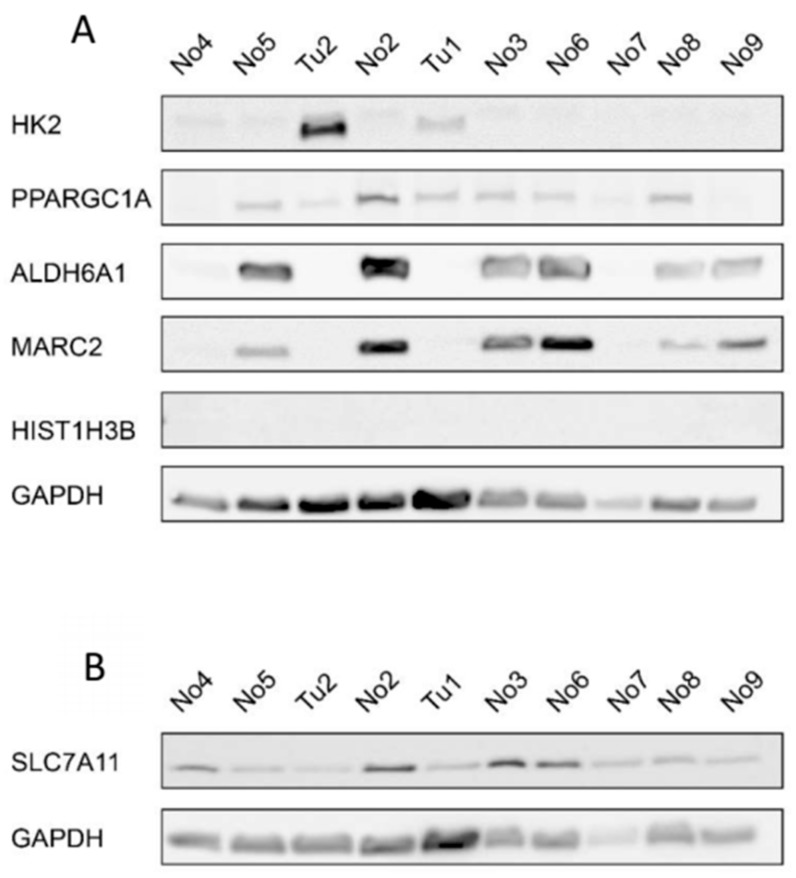
Protein expression of selected genes with deregulated expression in CDCs. (**A**) Western blot for HK2, PPARGC1A, ALDH6A1, MARC2, and HIST1H3B with GAPDH as the reference protein and (**B**) for SLC7A11 with GAPDH as the reference protein. Tu-tumor tissue sample; No-normal tissue sample.

To further validate the expression of the potential miRNA target genes, we also assessed the protein expression of the target genes by western blotting (Figure 4 and Figure S2). HK2 protein expression was increased in at least one CDC sample compared to the normal samples. HIST1H3B was not detectable in our samples and, unexpectedly, SLC7A11 protein expression was decreased in the CDC samples compared to the normal samples. As expected, PPARGC1A, ALDH6A1, and MARC2 protein expression was downregulated in the CDC samples compared to most of the normal samples.

### 2.6. Solute Carrier Genes

**Figure 5:**
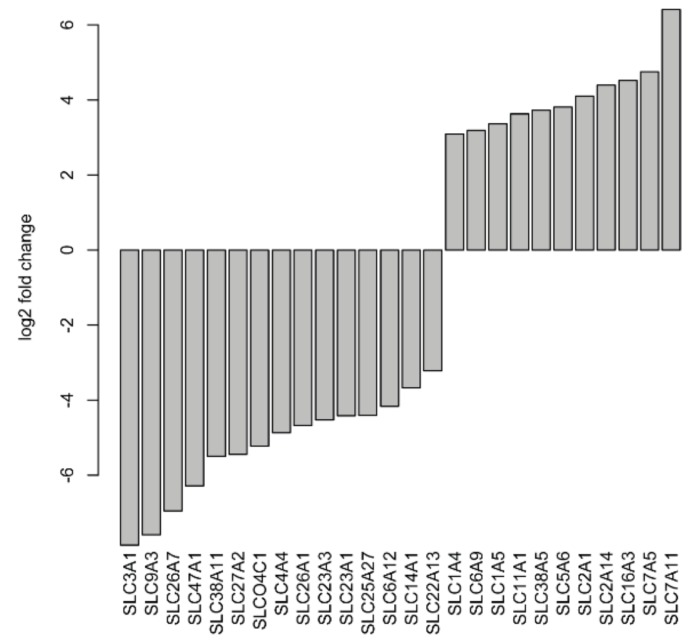
Deregulated solute carrier genes at |log2fold change| ≥3 in CDC samples compared to normal tissues.

As previously described, many SLC genes in CDC are dysregulated in comparison to normal tissue samples [8,11]. Therefore, we decided to investigate the expression of SLC genes in more detail. After applying a |log2fold change| ≥3 cutoff, a total of 15 SLC genes (*SLC3A1*, *SLC4A4*, *SLC6A12*, *SLC9A3*, *SLC14A1*, *SLC22A13*, *SLC23A1*, *SLC23A3*, *SLC25A27*, *SLC26A1*, *SLC26A7*, *SLC27A2*, *SLC38A11*, *SLC47A1*, and *SLCO4C1*) were found to be significantly downregulated, and 11 SLC genes (*SLC1A4*, *SLC1A5*, *SLC2A1*, *SLC2A14*, *SLC5A6*, *SLC6A9*, *SLC7A5*, *SLC7A11*, *SLC11A1*, *SLC16A3*, and *SLC38A5*) were significantly upregulated in CDC samples compared to normal samples (Figure 5). With even more stringent criteria of a |log2fold change| ≥6, four SLC genes (*SLC3A1*, *SLC9A3*, *SLC26A7*, and *SLC47A1*) were still significantly downregulated.

### 2.7. Survival Analysis of Deregulated Genes

**Figure 6:**
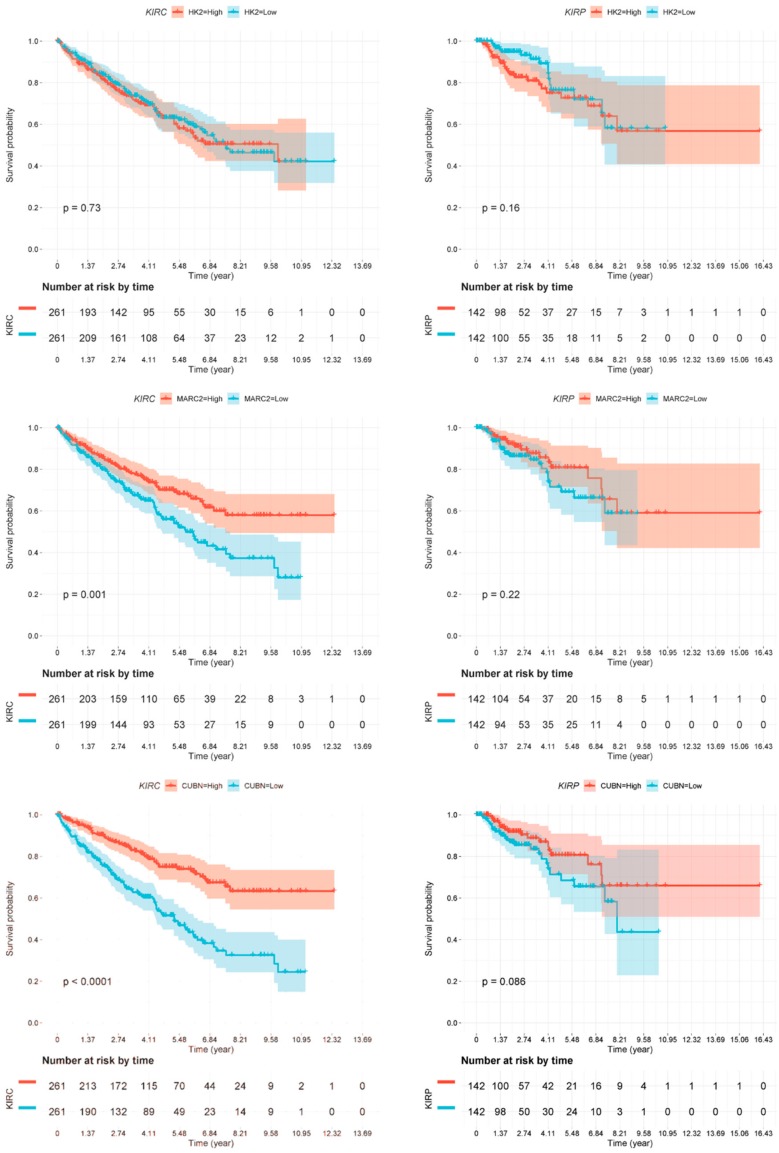

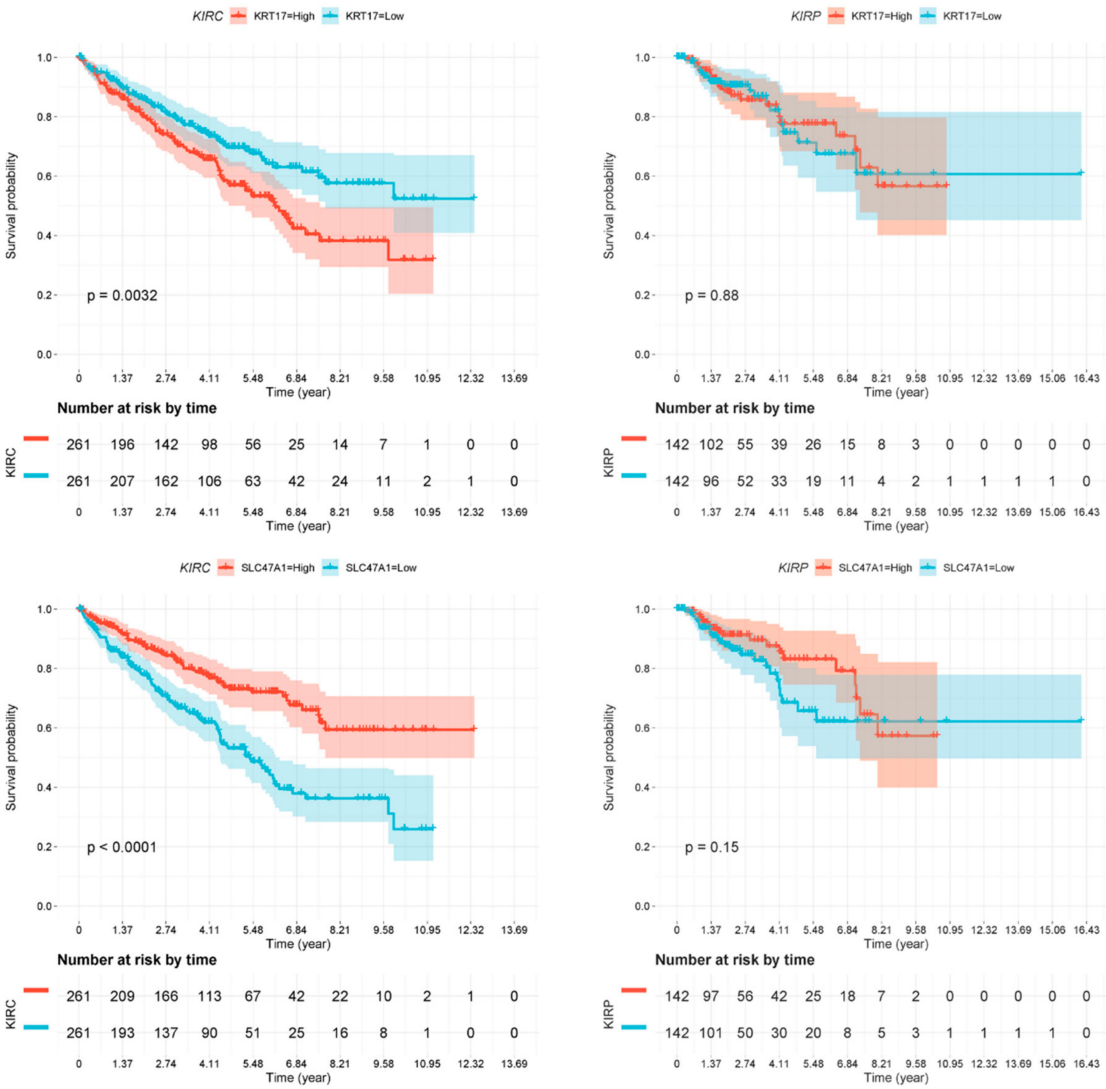
Kaplan-Meier analyses. Association of deregulated gene expression in CDCs with overall survival in clear cell renal cell carcinoma (ccRCC patients; KIRC dataset) and in papillary renal cell carcinoma (pRCC patients; KIRP dataset). Expression of none of the genes was significantly associated with overall survival in pRCC patients. However, the expression of all genes but *HK2* was significantly associated with overall survival, as shown for *HK2* (nonsignificant), *MARC2* (*p* = 0.001), *CUBN* (*p* ≤ 0.0001), *KRT17* (*p* = 0.0032), and *SLC47A1* (*p* ≤ 0.0001).

As we have described several distinct differences between CDC and normal tissue samples both in gene expression and in miRNA target gene expression, we sought to investigate whether these markers might provide any prognostic information. We used the TCGA dataset of renal cell carcinoma patients [17] and generated Kaplan-Meier analyses for the two most deregulated transcripts (*CUBN* and *KRT17*) as well as for the identified miRNA target genes. As the TCGA cohort did not contain any specified CDC patients, we performed this analysis independently for the two main histological subtypes of clear cell renal cell carcinoma patients (ccRCC; KIRC dataset) and for papillary renal cell carcinoma patients (pRCC; KIRP dataset) (Figure 6). The gene expression values, if available in the TCGA dataset, were separated at the median to generate a low-expression and a high-expression subgroup, which were analyzed for differences in patient survival.

Several of the genes identified by our approach were confirmed to be of prognostic relevance in the ccRCC patient cohort (KIRC). Low *MARC2* gene expression was significantly associated with poor overall survival (*p* = 0.001). Moreover, low expression of *CUBN* (*p* < 0.0001) and *SLC47A1* (*p* < 0.0001) and high expression of the gene *KRT17* (*p* = 0.0032) were significantly associated with poor overall survival (Figure 6). However, none of the genes analyzed were associated with overall survival in the pRCC (KIRP) patient cohort.

Survival analysis data of genes *PPARGC1A*, *ALDH6A1*, and *SLC7A11* in the same TCGA KIRC dataset have already been published by other authors and are therefore not repeated by us. Significant associations of low *PPARGC1A*, low *ALDH6A1*, and high *SLC7A11* gene expression with poor outcomes in ccRCC patients have been reported [8,16,17].

## 3. Discussion

CDC is a rare and highly aggressive variant of renal cell carcinoma, and is associated with a mean survival of approximately 11 months. The molecular pathways responsible for the tumor biology of CDCs are still mainly unresolved. As expected, after RNA transcriptome sequencing, we observed several cancer pathways and cell cycle regulation pathways that might predominantly be affected in CDCs compared to normal samples. Altogether, more genes were upregulated than downregulated in CDCs, which is in line with previous findings [6,8].

In our study, the most pronounced downregulated gene in CDC was *CUBN*. This gene is normally highly expressed in renal proximal tubules [12]. *CUBN* has not been previously described as deregulated in CDCs. However, we found a significant association of low *CUBN* gene expression with poor overall survival of ccRCC patients in the TCGA ccRCC dataset. In addition, this gene has already been identified as an independent prognostic marker for renal cell carcinoma at the protein level [18]. Interestingly, *CUBN* has been suggested as a predictive marker for the treatment of renal cancer patients with sunitinib and sorafenib [19]. So far, there are only case reports for CDCs treated with sunitinib or sorafenib, but there have been some promising results concerning partial responses [3].

In our study, the most pronounced upregulated gene in CDC was *KRT17*. *KRT17* is normally expressed in the basal cells of complex epithelia, but not in stratified or simple epithelia. Furthermore, it is an intermediate filament protein that is rapidly induced in wounded stratified epithelia and regulates cell growth by binding to the adaptor protein 14-3-3-sigma [13]. This finding is relevant to the consideration that “tumors are wounds that do not heal” [20]. *KRT17* expression is known to be associated with disease severity in oral submucosa fibrosis [14]. In line with this finding, *keratin 17* is induced in oral cancer and facilitates tumor growth [15]. Remarkably, Malouf and colleagues found in their functional enrichment analysis of CDC that response to wounding was the predominant pathway; however, when comparing CDCs with UTUCs, *KRT17* was among the top downregulated genes in CDC [6]. In our study, we observed a significant association between high *KRT17* gene expression and shorter overall survival in ccRCC patients in the KIRC dataset.

Solute carriers (SLCs) have been described as biomarkers for RCC patients [21,22]. Strikingly, SLC gene expression is changed in CDCs [8]. Wang and colleagues found several members of the SLC family among the top deregulated genes, either upregulated, i.e., *SLC6A11*, *SLC6A15*, *SLC7A3*, *SLCO1B1*, and *SLCO1B3* or downregulated, i.e., *SLC5A12*, *SLC12A1*, *SLC22A12*, *SLC47A2*, and *SLC22A6* [8]. In our study, four SLC transporter genes were strongly (|log2fold change| ≥6) downregulated (*SLC3A1*, *SLC9A3*, *SLC26A7*, and *SLC47A1*). *SLC7A11* was detected to be among the most upregulated at the RNA level in the study by Wang and colleagues [8]. We confirmed this gene as upregulated in one of two CDC samples. However, at the protein level, SLC7A11 was detected as downregulated in both CDCs compared to the normal samples. The observed discrepancy between the RNA and protein levels could be explained by post-transcriptional regulation of *SLC7A11*, since alternative 3’UTRs for *SLC7A11* have been described, but this has not been further studied [23]. In contrast to our findings, Wang et al. detected a protein upregulation of *SLC7A11* in 12 out of 15 CDC cases, and they stated that *SLC7A11* upregulation at the RNA level was associated with poor survival in ccRCC [8]. Their suggestion to target *SLC7A11* as a therapy option has to be, in our opinion, based on testing SLC7A11 protein expression on a case-by-case basis and needs further investigation. Wang et al. reported that two SLC members, *SLC47A2* and *SLC47A1*, were downregulated (|log2fold change| >6 and >5, respectively) in CDCs [8]. In line with this observation, we found that *SLC47A1* was also strongly downregulated at the RNA level (|log2fold change| ≥6). *SLC47A1* and *SLC47A2* are transporters that excrete endogenous and exogenous toxic electrolytes through urine and bile [24]. In addition, the *SLC47A* gene may affect renal excretion of substrate drugs, such as metformin [25]. It is tempting to speculate that treatment of tumors with a downregulated *SLC47A* gene, e.g., CDCs, with metformin could have toxic effects; however, polymorphisms in the *SLC47A* gene may affect renal excretion of substrate drugs such as metformin, resulting in inadequate pharmacotherapy or toxic effects [25]. Therefore, before considering the application of metformin in tumor patients, these somatic polymorphisms should be tested. Notably, our two CDC patients did not possess single nucleotide variants in the *SLC47A* gene.

We utilized RNA sequencing information about pre-miRNAs or miRNA host genes as an alternative approach to identify target genes or proteins deregulated in CDC. In this way, we identified strong correlations between downregulated miR-374b-5p and its upregulated target genes *HIST1H3B*, *HK2*, and *SLC7A11*, and also between upregulated miR-26b-5p and its downregulated target genes *PPARGC1A*, *ALDH6A1*, and *MARC2*. Among the upregulated target genes, *HIST1H3B* has not yet been described to play any role in renal cell carcinomas. HK2 is well known as an enzyme in glycolysis that catalyzes the phosphorylation of glucose into glucose-6-phosphate [26]. HK2 has been described as a target of the HIF1a protein in several cancers, including RCC [27,28]. Recently, Nam et al. showed that HK2 plays a pivotal role in renal tumor progression to metastasis [29]. SLC7A11 (xCT) is an anionic amino acid transporter that is highly specific for the amino acids cysteine and glutamate [30]. Increased expression of *SLC7A11* at the RNA and protein levels in CDCs has been shown previously [8,11].

In our set of downregulated genes, *PPARGC1A* (*PGC-1α*) is a central regulator of mitochondrial energy metabolism and functions in renoprotection against ischemia [31]. LaGory and coworkers found that ccRCC cells expressing *PGC-1α* showed impaired tumor growth and enhanced sensitivity to cytotoxic therapies [32]. In line with this, RCC patients with low levels of *PGC-1α* expression displayed a poor outcome in the TCGA ccRCC dataset [32]. *ALDH6A1* catalyzes the oxidative decarboxylation of malonate and methylmalonate semialdehydes to acetyl- and propionyl-CoA in the valine and pyrimidine catabolic pathways [33]. Recently, Zhang et al. identified six genes, including *ALDH6A1*, as biomarkers for ccRCC, and demonstrated that downregulation of the *ALDH6A1* gene was associated with shorter overall survival of ccRCC patients in the TCGA dataset [34]. *MARC2* (*MOSC2*) has been suggested to play a role in the mitochondrial nitric oxidase pathway and in the detoxification of xenobiotics [35]. MARC2 associates with MARC1 in the mitochondrial amidoxime-reducing component (mARC), i.e., mammalian molybdenum-containing enzymes [35]. Rixen and coworkers recently showed that *MARC2* KO mice had decreased levels of total cholesterol and increased glucose levels, suggesting that *MARC2* affects energy pathways [36]. However, Li et al. showed that reduced *MARC2* expression was associated with an increased sensitivity to paclitaxel-based neoadjuvant therapy in human *EGFR-2*-negative breast cancer patients [37] but, to the best of our knowledge, there have been no previous reports on a role of *MARC2* in RCC.

In our survival analysis, we showed for the first time that low gene expression for *MARC2*, *CUBN*, and *SLC47A1* and high gene expression of *KRT17* were associated with poor overall survival of ccRCC patients. The limitations of our study were the small number of CDCs studied and the lack of an available validation set. The strength of our study is that we considered miRNA-mRNA correlations and could further confirm the role of SLC in CDCs. Based on the finding that among the deregulated genes in CDCs were genes that regulate (i) the transport of amino acids or electrolytes (*SLC7A11*, *SLC47A1*), (ii) mitochondrial pathways (*MARC2*, *PPARGC1A*), and (iii) catabolic pathways (*HK2*, *ALDH6A1*), we can support the statement that CDC is a metabolic disease [6].

## 4. Material and Methods

### 4.1. Patients and Tumor Material

The snap-frozen tissue samples were obtained from the Comprehensive Cancer Center tissue biobank of the University Hospital Erlangen. The tumor histology was reviewed by experienced uropathologists (AH and FH). All procedures were performed in accordance with the ethical standards established in the 1964 Declaration of Helsinki and its later amendments. All patients gave informed consent. The study was based on the approval of the Ethics Commissions of the University Hospital Erlangen (No. 4607). The CDC case Tu1 (pT3a, pN2, G3–G4) and CDC case Tu2 (pT3a, cN2, cM1, G3) presented with liver metastases at diagnosis and had a survival time of 2 months. The normal tissue samples originated in one case (No2) from CDC Tu2 adjacent tissue, in five cases from tumor-adjacent tissues from ccRCC patients (No3, No4, No7, No8, No9), in one case from tumor adjacent tissue from a chromophobe renal cell carcinoma patient (No5), and in one case from tumor adjacent tissue from an oncocytoma patient (No6) (Table S1).

### 4.2. RNA and Protein Isolation

Total RNA and protein were isolated using TRIzol (Invitrogen, Darmstadt, Germany) according to the manufacturer’s instructions. Tissue samples were mechanically disrupted in TRIzol reagent prior to RNA and protein isolation. RNA preparations were treated with recombinant DNase I (Sigma Aldrich, Taufkirchen, Germany) before use. The RNA yield and purity were determined using a microliter spectrophotometer (NanoDrop 1000, Thermo Fisher Scientific, Wilmington, DE, USA).

### 4.3. Quantitative Real-Time PCR

The mRNA transcripts were detected using TaqMan gene expression assays (Thermo Fisher Scientific) according to the manufacturer’s protocol. Briefly, 1 µg of RNA was reverse transcribed using the Maxima cDNA synthesis kit (Thermo Fisher Scientific). The reactions were carried out using the StepOne Plus Real-Time PCR System (Thermo Fisher Scientific) in triplicate in a final volume of 10 µL with cDNA equivalent to 25 ng RNA, using TaqMan gene expression assays (SLC7A11, Hs00921938_m1; HIST1H3B, Hs00605810_s1; HK2, Hs00606086_m1; PPARGC1A, Hs00173304_m1; ALDH6A1, Hs00194421_m1; MARC2, Hs01550747_m1; SLC47A1, Hs00217320_m1) and PCR reagents according to the manufacturer’s instructions. No template controls were included in the reaction plates. Thermal cycling conditions were 95 °C for 20 s, followed by 40 cycles of 95 °C for 1 s and 60 °C for 20 s. GAPDH (Hs99999905_m1, Thermo Fisher Scientific) served as the endogenous reference. Relative mRNA expression levels were calculated according to the ∆∆Ct method [38].

### 4.4. Western Blotting

Twenty-five µg of protein extract was separated by SDS-PAGE (8% gels) and transferred to nitrocellulose membranes (GE Healthcare, Freiburg, Germany) by semidry electroblotting. The primary antibodies used were against *SLC7A11* (rabbit mAb, clone *D2M7A*, 1:1000, Cell Signaling, Frankfurt, Germany), *HIST1H3B* (rabbit pAb, PA5-111876, 1:1000, Thermo Fisher Scientific), *HK2* (rabbit mAb, clone C64G5, 1:1000, Cell Signaling), *PPARGC1A* (mouse mAb, clone 1C1B2, 1:3000, Proteintech, Manchester, UK), *ALDH6A1* (rabbit pAb, 20452-1-AP, 1:6000, Proteintech), *MARC2* (rabbit pAb, 24782-1-AP, 1:1000, Proteintech), *SLC47A1* (rabbit mAb, clone D4C62, 1:500, Cell Signaling), and GAPDH (rabbit mAb, clone 14C10, 1:10,000, Cell Signaling). Secondary horseradish peroxidase-conjugated antibodies against rabbit or mouse were purchased from Jackson ImmunoResearch (Suffolk, UK) and used at a concentration of 1:5000. Protein bands were detected by enhanced chemiluminescence in an LAS-4000 chemiluminescence detection system (GE Healthcare, Munich, Germany).

### 4.5. RNA Sequencing Data Processing

Total RNA sequencing library preparation and sequencing was performed at Core Facility Genomik (University of Münster, Münster, Germany). After rRNA depletion (NEBNext; New England Biolabs, Ipswich, MA, USA), library preparation was performed according to the manufacturer’s protocols (NEBNext Ultra II, New England Biolabs). RNA sequencing was performed using the Illumina NextSeq 500 platform. Processing of 75 bp single-end reads of mRNA sequence data was quality checked using FastQC (v 0.11.8) [39]. Low-quality read ends and remaining sequencing adapters were clipped off using Cutadapt (v1.14) (https://cutadapt.readthedocs.io/en/stable/). Trimmed reads were aligned to the human genome (UCSC GRCh38) using HiSat2 (v2.1.0) (https://ccb.jhu.edu/software/hisat2/index.shtml). Annotation and counting of the processed reads was performed using featureCounts (v 1.5.3) (http://subread.sourceforge.net/) and Ensembl annotations (90, GRCh38.p10). Mapping results can be assessed in Table S6. Read counts of all genes can be found in Table S7.

### 4.6. Differential Gene Expression Analysis

Differential gene expression analysis was performed in R using DESeq2 v1.16.1 [40]. We first filtered genes by keeping those with at least five read counts in at least three normal tissues, and at least five read counts in both tumor samples. As a result, 16,672 out of 58,395 genes were used for follow-up analysis (Figure S1). Finally, we used an algorithm to estimate variance-mean dependence in read counts and test for differential expression based on a model using a negative binomial distribution. The Benjamini-Hochberg correction was used to correct for multiple comparisons. Genes with an adjusted *p*-value ≤ 0.05 were regarded as significantly differentially expressed. Statistics of the differential gene expression results including base mean, fold-change, and adjusted *p*-values of genes were visualized in a circos plot using OmicCircos. Hierarchical clustering of samples was performed and visualized using ComplexHeatmap [41].

### 4.7. Gene Enrichment Analyses

Significantly differentially expressed protein-coding genes with at least a 2-fold increase or a half-fold change were used to perform gene enrichment analysis using Enrichr [42]. This tool applies Fisher’s exact test to determine whether a given set of genes is significantly associated with curated biological pathways from databases such as KEGG [43], WikiPathways [44], or Reactome [45]. The Benjamini-Hochberg correction was used to correct for multiple comparisons. The pathways with adjusted *p*-values ≤ 0.05 were regarded as significant. The results can be found in Table S2.

### 4.8. Survival Analysis

We extracted RNA sequencing data from 522 clear cell renal cell carcinoma (KIRC) patients and 284 papillary cell renal cell carcinoma (KIPR) patients from the TCGA database [17]. Patients were divided into two groups (high or low) based on their expression levels of the genes of interest (i.e., *HK2*, *MARC2*, *CUBN*, *KRT17*, *SLC47A1*). Patients at the top 50% expression level of a gene were assigned to the high group, and the other patients were assigned to the low group. Patient survival times were calculated as the number of days from initial pathological diagnosis to death, or the number of days from initial pathological diagnosis to the last time the patient was known to be alive. These times were used to generate the Kaplan-Meier survival plots using RTCGA (https://rtcga.github.io/RTCGA).

### 4.9. miRNA Target Genes

To derive miRNA-gene interactions, we combined results from three databases. We first obtained predictive miRNA-gene interactions from TargetScan v7.2 [46]. The data were further annotated with StarBase v2.0 [47] and miRTarbase 2018 [48], which provide experimental evidence for the putative miRNA-gene interactions. As a result, we obtained a list of miRNA-gene interactions that not only contained putative miRNA binding sites in 3’ UTR of target genes, but also experimental evidence validating such interactions. The list can be found in Table S4.

## 5. Conclusions

The RNA sequencing analysis of CDCs in comparison to normal tissues revealed a large number of dysregulated protein-coding genes with a predominance of solute carrier transporters, potential miRNA-target interactions and prognostic markers that could be associated with CDC.

## Figures and Tables

**Table 1:** Computational correlation analysis of miRNAs and their target genes.

miRNA	Target Gene	Correlation Coefficient	*p*-Value	log2fold Change of Target Genes	qRT-PCR of Target Genes
miR-374b-5p	*SLC7A11*	−0.67	0.034	6.41	up
miR-374b-5p	*HIST1H3B*	−0.71	0.021	5.87	up
miR-374b-5p	*HK2*	−0.74	0.013	5.72	up
miR-26b-5p	*PPARGC1A*	−0.70	0.020	−4.87	down
miR-26b-5p	*ALDH6A1*	−0.66	0.039	−4.71	down
miR-26b-5p	*MARC2*	−0.68	0.030	−4.08	down
miR-26b-5p	*SLC7A11*	+0.82	0.004	6.41	up

## Supplementary Materials

The following are available online at https://www.mdpi.com/2072-6694/12/1/64/s1, Figure S1: Analysis of the expression of the selected 16,672 genes, Figure S2: Western blots and densitometry, Table S1: Results of differential gene expression analysis. The table contains four sub-tables that are statistics of all selected genes (*n* = 16,672), significantly (adjusted *p*-value ≤ 0.05) differentially expressed protein-coding genes with |log2fold-change ≥1|, |log2fold-change ≥3| and |log2fold-change ≥6|, Table S2: Results of gene set enrichment analysis, Table S3: RNA transcriptome sequencing data for single nucleotide variations, Table S4: miRNA-gene interactions and correlation analysis, Table S5: Putative miRNA binding sites on 3’ UTR of *SLC7A11*, Table S6: Statistics of the read counts that were mapped and identified in the RNA sequencing data, Table S7: Read counts of all identified genes (*n* = 58,396).

## Abbreviations

CDC	collecting duct renal cell carcinoma
SLC	solute carrier
SLC7A11	solute carrier family 7, member 11
SLC47A1	solute carrier family 47, member 1
