# Supplementary material for: RNA Sequencing of Collecting Duct Renal Cell Carcinoma Suggests an Interaction between miRNA and Target Genes and a Predominance of Deregulated Solute Carrier Genes
Source: Cancers (Basel). 2019 Dec 24;12(1):64. doi: 10.3390/cancers12010064 (PMC7017122; doi:10.3390/cancers12010064)
Supplement: Supplementary file 1 [file cancers-12-00064-s001.zip › cancers-667778-suppl-XML/cancers-667778-suppl-Figures.docx]

Supplementary Materials: RNA Sequencing of Collecting Duct Renal Cell Carcinoma Suggests an Interaction between miRNA and Target Genes and a Predominance of Deregulated Solute Carrier Genes

Sven Wach, Helge Taubert, Katrin Weigelt, Nora Hase, Marcel Köhn, Danny Misiak, Stefan Hüttelmaier, Christine G. Stöhr, Andreas Kahlmeyer, Florian Haller, Julio Vera, Arndt Hartmann, Bernd Wullich and Xin Lai


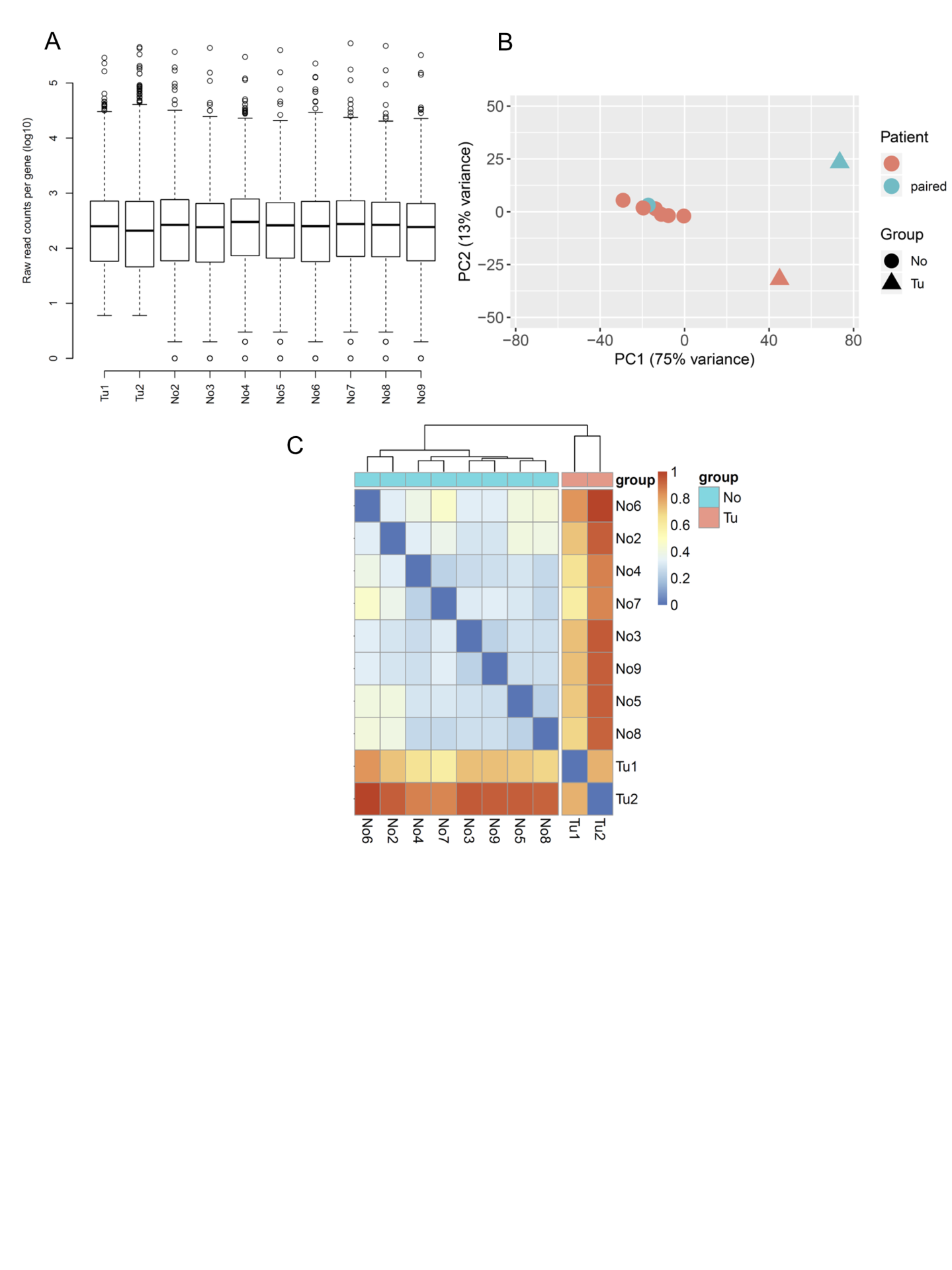


**Figure S1.** Analysis of read counts of the selected 16672 genes. (**A**) Distribution of read counts of the selected genes. The upper and lower whiskers represent minimum and maximum values of read counts (in log10 scale), respectively. The lower, middle, and upper bands of the box represents 1st (25%), 2nd (50%) and 3rd (75%) quartile of the data, respectively. The circles represent outliers. (**B**) Principal component analysis. The read counts of the selected genes were first transformed to the log2 scale and normalized with respect to library size, and then used for the analysis. The first two principal components take account for 88% variance of the data. The samples were classified by shapes. The paired samples were highlighted in cyan. (**C**) Samples clustering. The Euclidian distance between samples was calculated using the selected genes and the distance were used for clustering the samples. Values of distance were normalized to the maximum distance between samples.


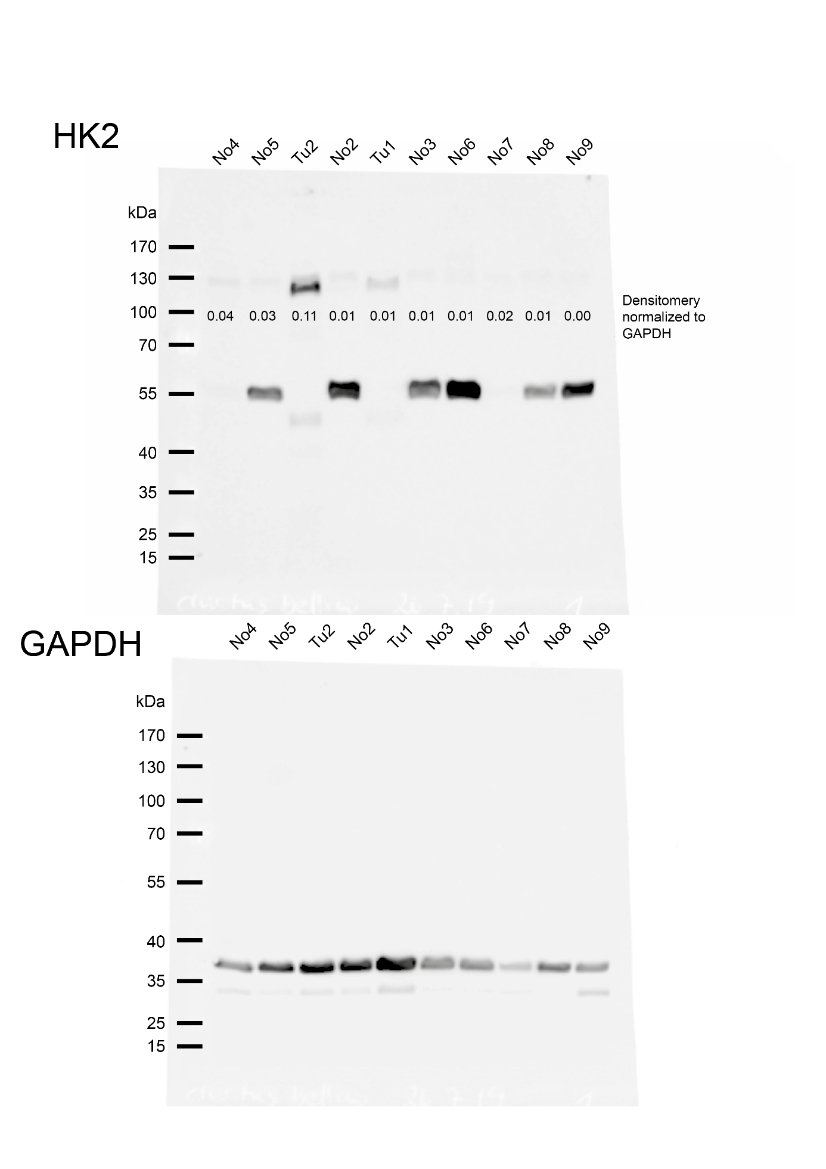

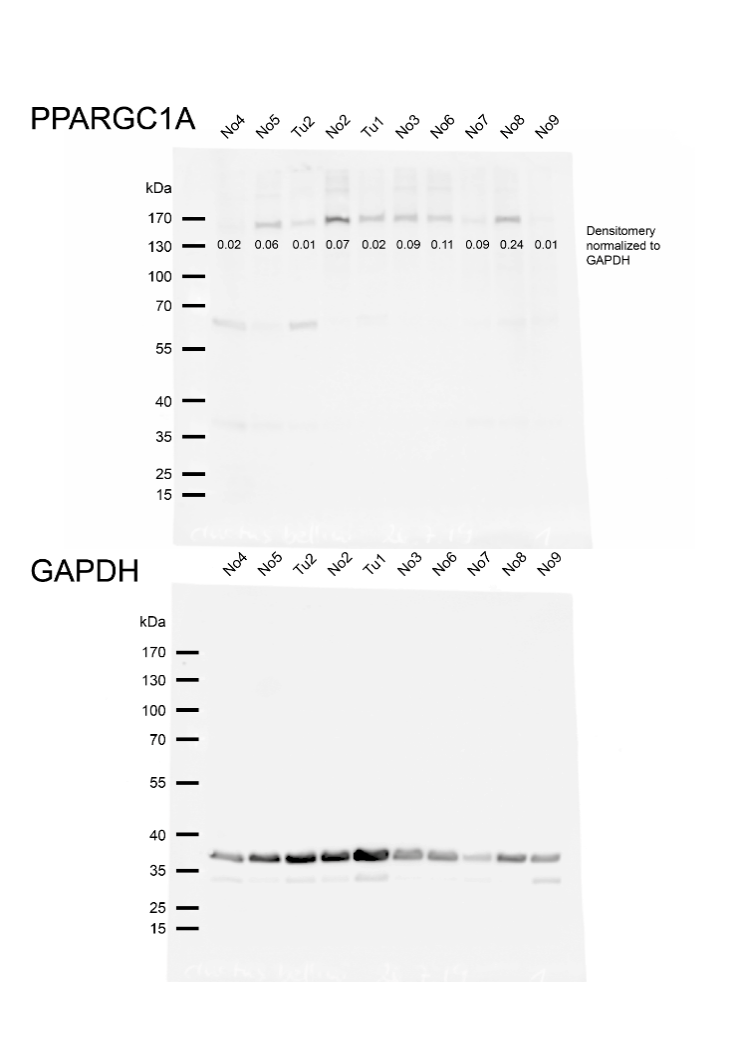


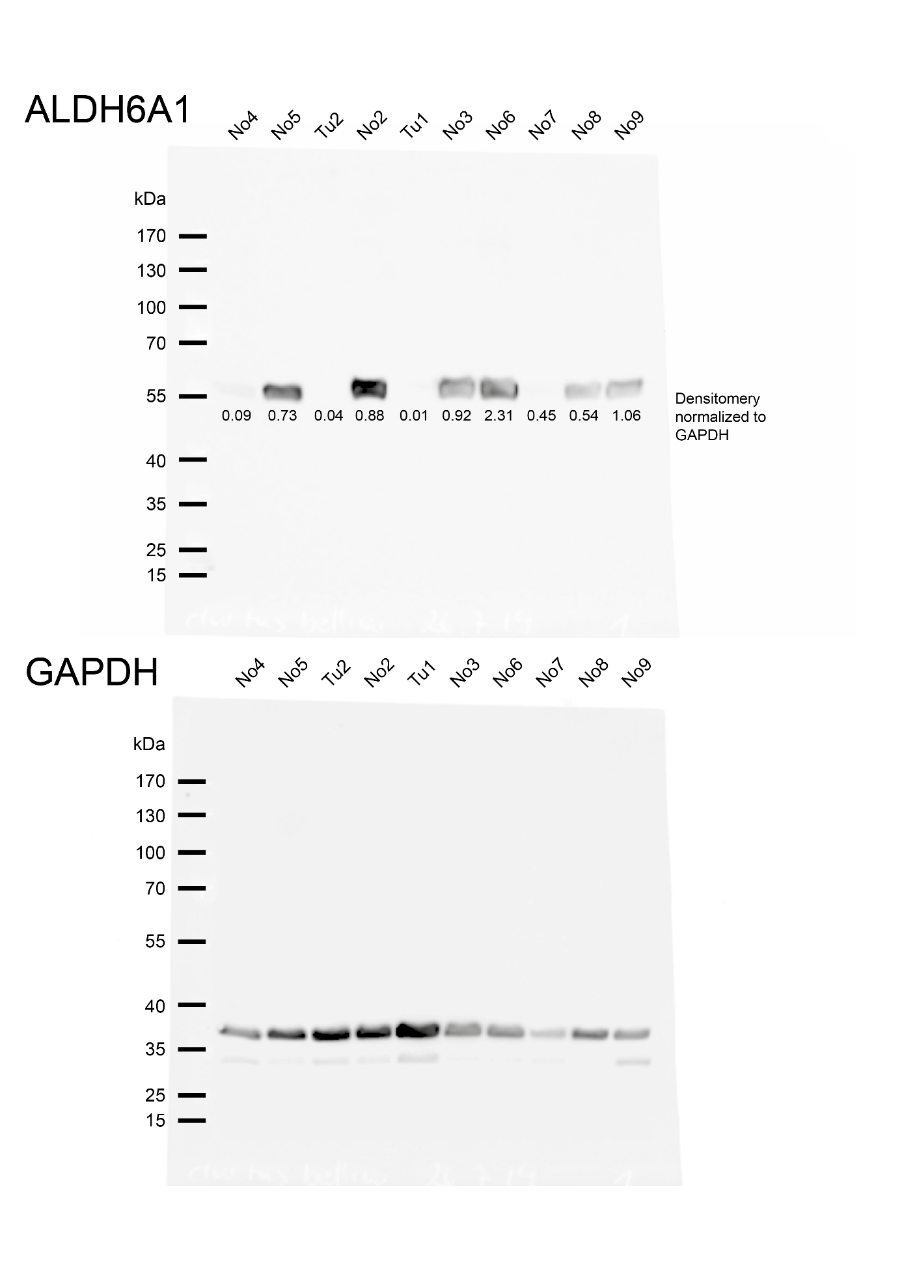

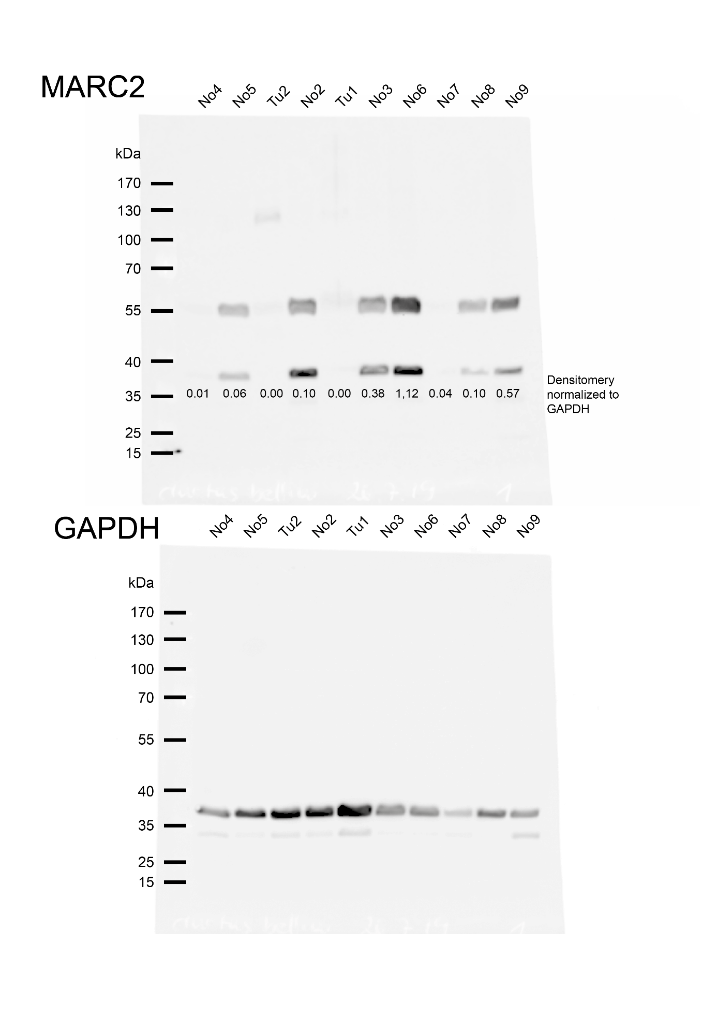


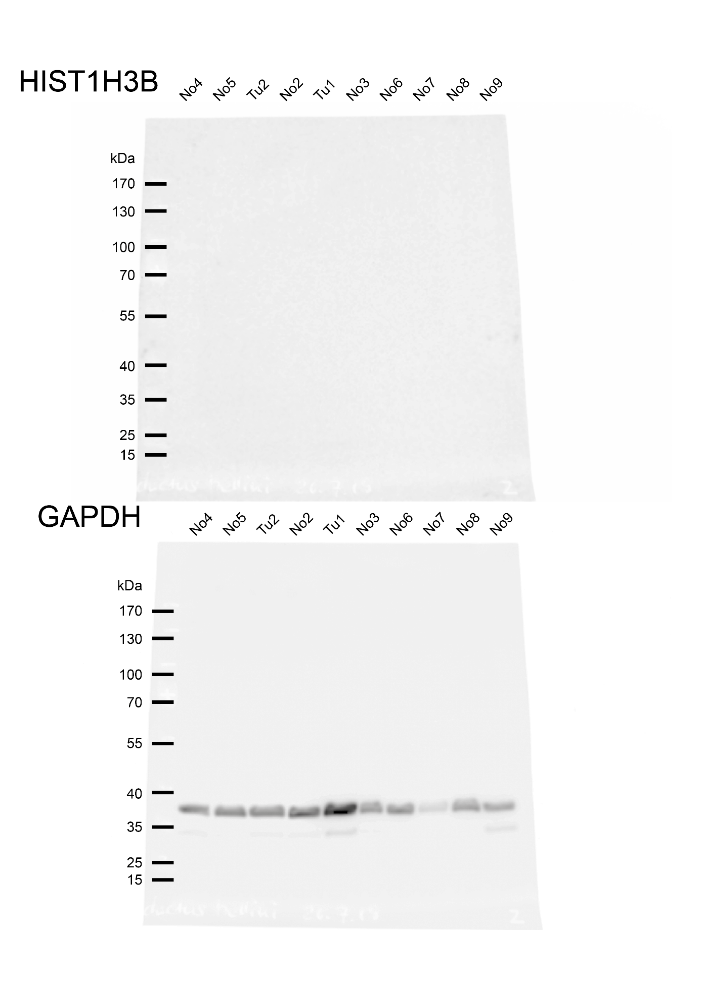

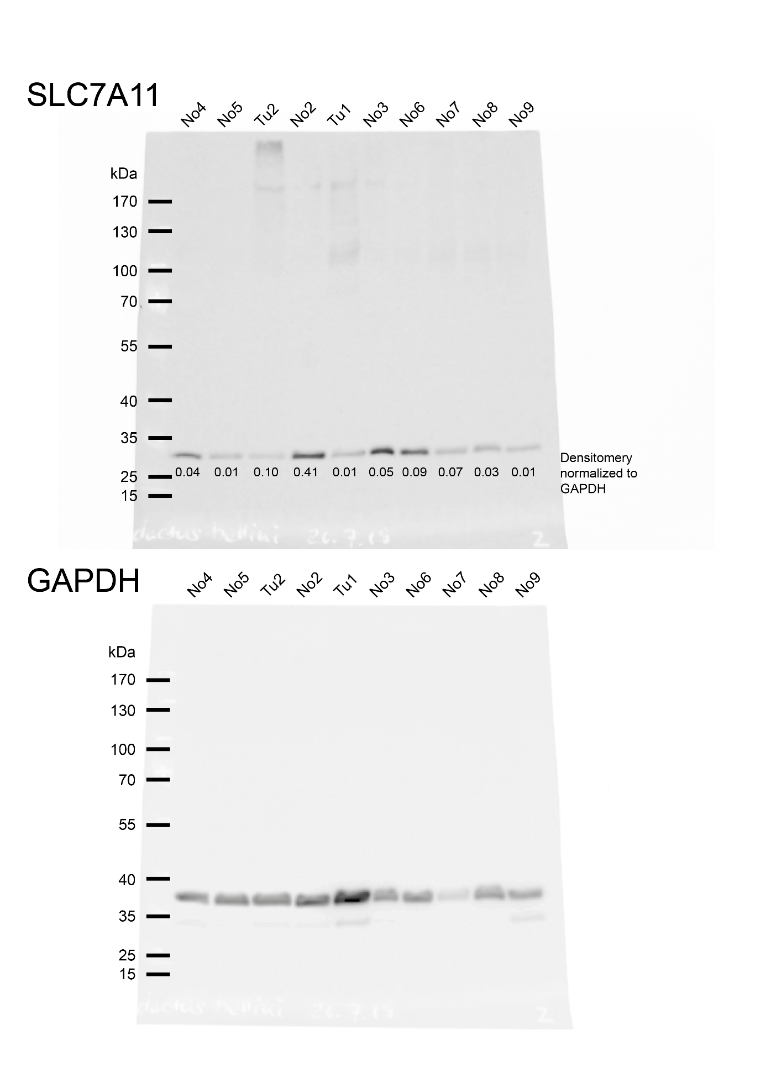


**Figure S2.** Western blots and densitometry. The Western blots for the proteins (HK2, PPARGC1A, ALDH6A1, MARC2, HIST1H3B and SLC7A11) and their densitometric analysis (protein/GAPDH as reference protein) are given. Densitometric analysis appears below the relevant bands for the protein. Please, note that Western blots were performed but for SLC7A11 on the same blot. Therefore, bands from previous protein blots can also appear on other blots. The GAPDH blot is for all proteins the same but for SLC7A11 were a new gel/blot was performed including a blot for GAPDH.

| 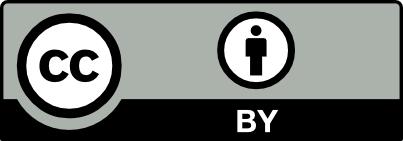 | © 2020 by the authors. Licensee MDPI, Basel, Switzerland. This article is an open access article distributed under the terms and conditions of the Creative Commons Attribution (CC BY) license (http://creativecommons.org/licenses/by/4.0/). |
| --- | --- |
